# Circadian Effects on Vascular Immunopathologies

**DOI:** 10.1161/CIRCRESAHA.123.323619

**Published:** 2024-03-15

**Authors:** Qun Zeng, Valeria Maria Oliva, María Ángeles Moro, Christoph Scheiermann

**Affiliations:** Department of Pathology and Immunology, Faculty of Medicine, University of Geneva, Geneva, Switzerland (Q.Z., V.M.O., C.S.).; Centro Nacional de Investigaciones Cardiovasculares Carlos III, Madrid, Spain (M.Á.M.).; Geneva Center for Inflammation Research, Switzerland (C.S.).; Translational Research Centre in Oncohaematology, Geneva, Switzerland (C.S.).; Biomedical Center, Institute for Cardiovascular Physiology and Pathophysiology, Walter Brendel Center for Experimental Medicine, Faculty of Medicine, Ludwig-Maximilians-Universität Munich, Germany (C.S.).

**Keywords:** atherosclerosis, circadian rhythm, endothelial cells, infarction, leukocytes

## Abstract

Circadian rhythms exert a profound impact on most aspects of mammalian physiology, including the immune and cardiovascular systems. Leukocytes engage in time-of-day–dependent interactions with the vasculature, facilitating the emigration to and the immune surveillance of tissues. This review provides an overview of circadian control of immune-vascular interactions in both the steady state and cardiovascular diseases such as atherosclerosis and infarction. Circadian rhythms impact both the immune and vascular facets of these interactions, primarily through the regulation of chemoattractant and adhesion molecules on immune and endothelial cells. Misaligned light conditions disrupt this rhythm, generally exacerbating atherosclerosis and infarction. In cardiovascular diseases, distinct circadian clock genes, while functioning as part of an integrated circadian system, can have proinflammatory or anti-inflammatory effects on these immune-vascular interactions. Here, we discuss the mechanisms and relevance of circadian rhythms in vascular immunopathologies.

Circadian rhythms exhibit a ≈24-hour (circa diem) cycle. They represent oscillations present in most cells, even in red blood cells lacking a nucleus.^1,2^ These rhythms influence most physiological aspects, including cardiovascular functions and the immune system.^3,4^ Circadian rhythms are intrinsic to an organism, which means that they persist in constant conditions, as present, for example, in continuous darkness. However, they can be synchronized to changing environments (eg, when traveling to other time zones) by external cues such as light.^5^ Mammals have a central circadian pacemaker that aligns the body to the environment. It sits in the suprachiasmatic nucleus (SCN) of the hypothalamus, where it receives light entrainment cues and subsequently synchronizes circadian clocks throughout the body via neurohumoral means.^1^

Mammalian circadian clocks rely on highly interconnected transcription-translation feedback loops, driven by the rhythmic expression and function of circadian clock genes. *BMAL1* (brain and muscle aryl hydrocarbon receptor nuclear translocator-like 1) is one of the most important and studied circadian clock genes because it is the only single clock gene whose absence in itself abrogates general rhythmicity.^6^ *BMAL1* heterodimerizes with the protein product of another clock gene, aptly named circadian locomotor output cycles kaput (*CLOCK*), to form the transcription factor complex *BMAL1*:*CLOCK*. Together, they activate the transcription of clock-controlled genes by binding to the enhancer (E)-box region (CACGTG) within their respective promoter regions.^7,8^ Repressor circadian clock genes *Period* (*PER1/2/3*) and *Cryptochrome* (*CRY1/2*) counterbalance this activation, creating a self-sustaining 24-hour cycle.^7,8^ Additional feedback loops involving the transcription factors, retinoic acid receptor-related orphan receptor α and REV-ERBα/β (reverse strand of ERBα/β; encoded by *NR1D1/2*), further fine-tune the clock by activating and suppressing the transcription of *BMAL1*, respectively.^7–9^ While transcription-translation feedback loops are governing circadian rhythms in cells of both the cardiovascular and the immune system, erythrocytes use oscillations in their redox state to maintain circadian functions.^10^

Circadian rhythms are thought to have evolved in anticipation of regularly recurring events across the day, such as food availability and potential danger. The body’s primary defense against various threats, such as infections or harmful stimuli, is the immune system, which is strongly rhythmic.^4,11–18^ A key feature of the immune system is its high motility,^12^ with leukocytes trafficking through blood, organs, and lymph to survey the state of the body. Immune cells are, thus, intricately reliant on interactions with blood vessels to exert their functions. Leukocytes emigrate from the blood into tissues by a process termed the leukocyte adhesion cascade. This sequence of events begins with the capture of leukocytes and their rolling along the endothelial cell (EC) wall, followed by the induction of firm adhesion, intraluminal crawling, and transmigration from the bloodstream through the endothelial barrier into the tissue.^19^ These leukocyte-EC interactions play an important role in multiple cardiovascular diseases and are strongly circadian in nature. In this review, we will first briefly recapitulate circadian rhythms in the vasculature and immune cells. We will then discuss the impact of circadian rhythms on the crosstalk between immune cells and the vasculature in the steady state, as well as in cardiovascular diseases, focusing on atherosclerosis and infarction.

## CIRCADIAN RHYTHMS OF NONHEMATOPOIETIC CELLS OF THE CARDIOVASCULAR SYSTEM

Circadian rhythms are important regulators of vascular physiology and pathology, ranging from blood pressure^20^ and angiogenesis^21,22^ to atherosclerosis^20^ and infarction.^23^ Rhythmic expression of circadian clock genes has been described in many cell types of the cardiovascular system, such as ECs,^24–26^ smooth muscle cells (SMCs),^24,25^ and cardiomyocytes.^27^ Heart and vasculature resident macrophages play an important role in regulating cardiovascular function and are discussed in the section below. In the mouse aorta, microarray analyses of 6- to 8-week-old mice, sampled every 4 hours across 2 days, revealed that 330 genes (≈5% of detected genes) were oscillating in a circadian manner. These genes are related to protein dynamics, metabolism, vascular integrity, and response to injury.^28^ In agreement with these findings, bulk RNA sequencing of 7-week-old mouse aorta and heart, harvested every 2 hours across 2 days, revealed 4% and 6%, respectively, of protein-coding genes to be oscillatory.^29^ These data indicate that cardiovascular physiology is affected by circadian rhythms in gene expression. We briefly discuss the key circadian features of cells within the cardiovascular system; the reader is referred to several more specialized reviews on this subject for further details.^30,31^

### ECs

Circadian rhythms control the function of ECs. In healthy mice, RNAs of *Vegfa* (vascular endothelial growth factor A) and its 2 receptors, Vegfr1 (encoded by *Flt1*) and Vegfr2 (encoded by *Kdr*), oscillate in the heart, with *Vegfa* and *Kdr* (the main drivers of angiogenesis) peaking at dawn, while *Flt1* peaks at night.^29^ A similar oscillation of vascular endothelial growth factor (VEGF) proteins (≈2-fold) has also been observed in the plasma of tumor-bearing mice,^32,33^ with a peak of VEGF levels at Zeitgeber time (ZT) 2 to 6 (ie, 2–6 hours after light onset, in a 12-hour light:12-hour dark environment) and a trough at ZT14.^32^ Luciferase reporter gene analyses revealed that *Per2* and *Cry1*, which peaked at ZT14, inhibited the hypoxia-induced VEGF transcription.^32^ In addition, the knockdown of *Bmal1* suppressed angiogenesis through the blockade of EC cycle progression and, thus, proliferation.^22^ Furthermore, the endothelium of *Per2* mutant mice exhibits impaired relaxations to acetylcholine and ionomycin.^34^ Because ECs serve as gatekeepers at the blood-tissue interface, circadian oscillations in this cell type furthermore greatly impact leukocyte chemoattraction, adhesion and infiltration, and associated damage in cardiovascular complications such as infarcts.^35,36^ This is discussed in detail in the following section.

### Smooth Muscle Cells

SMC-specific deletion of *Bmal1* in mice (*SM22α^cre^:Bmal1^flox^*) led to diminished rhythms in blood pressure in constant darkness but not under normal light-dark cycles.^37^ In isolated mesenteric arteries, SMC-specific deletion of *Bmal1* was shown to abolish the time-of-day variation in contractile responses.^37^ Human aortic SMCs with *BMAL1* overexpression exhibit a fibroblast-like phenotype, while *BMAL1* knockdown suppressed this phenotype transition.^38^ In vivo, vascular SMC-specific *Bmal1* deletion (*Smmhc^creERT2^:Bmal1^flox^*) aggravated lesions in mouse atherosclerosis (≈2-fold) and enhanced SMC migration and apoptosis.^38,39^ These data implicate circadian oscillations in SMCs to be important in the regulation of blood pressure and atherosclerosis.

### Cardiomyocytes

The rhythmicity of the heartbeat is under the control of the autonomic nervous system. However, daily rhythms of heart electrical activities have been shown to be controlled by the balance of circadian inputs from the brain and the heart itself.^40^ Rat myocytes isolated at ZT15 (ie, their behavioral active phase) are more prone to arrhythmic activity in response to the sympathetic agonist isoproterenol compared with ZT3 (their behavioral rest phase) myocytes.^41^ Human cardiomyocytes derived from embryonic stem cells with *BMAL1* deficiency displayed features of dilated cardiomyopathy, showcasing reduced (≈2-fold) contractility, calcium dysregulation, and disarrayed myofilaments.^42^ Furthermore, accumulating evidence indicates that cardiomyocyte death, a major component of myocardial infarction (MI), is also under the control of circadian rhythms.^43^ Given the exquisite sensitivity of cardiomyocytes to autonomous nervous system control, the strong oscillations in neural activity greatly impact cardiomyocyte physiology and heart contractility across the day. Together, these examples show a significant impact of circadian rhythms on the function of major cell types in the cardiovascular system.

## CIRCADIAN RHYTHMS IN THE IMMUNE SYSTEM

Rhythmicity in immune responses was initially observed in the innate immune system in 1960,^44^ followed by findings in adaptive immune responses a decade later.^45^ Circadian gene expression has been detected in all subsets of leukocytes, regulating not only their distribution but also their differentiation and function.^13^ The most obvious oscillations in the immune system are fluctuations in blood leukocyte counts, with a peak occurring at the behavioral rest of the organism and a trough at the behavioral active phase.^46,47^ Thus, for diurnal humans, leukocytes peak at night,^46^ while in nocturnal rodents, this is observed at midday.^47^ In humans, studies showed fluctuations in the numbers of leukocyte subtypes such as neutrophils (1.5–2-fold), lymphocytes (≈1.5–2-fold), eosinophils (≈1.5–2-fold), or monocytes (≈1.3–2-fold).^48–50^ In mice, these oscillations are even greater (≈2–7-fold)^49^ likely due to less genetic variability and controlled environmental conditions. These oscillations greatly dictate the distribution of immune cells throughout the body, namely, in blood or tissues. This impacts, for example, the ability of lymphocytes and dendritic cells (DCs) to functionally interact in lymph nodes (LNs).^51^ As for the circadian regulation of general leukocyte functions, we only briefly touch on key findings in specific leukocyte subsets here; the reader is again referred to specialized reviews on the subject for further details.^12,13^

### Macrophages

Macrophages in the cardiovascular system are either tissue-resident or recruited as monocytes from blood, followed by their differentiation into macrophages. The latter is predominantly the case in cardiovascular disease, discussed in the following section. Macrophages from young mice exhibit rhythmic phagocytic activities, which peak at ZT12, while macrophages from aged mice lost this rhythmicity.^52^ Furthermore, *Bmal1*-deficient macrophages (*Lyz2^cre^:Bmal1^flox^*) exhibit enhanced phagocytic capacity, attributed to changes in their actin cytoskeletal organization.^53^ Circadian clock genes also modulate inflammatory processes in macrophages.^54^ For example, BMAL1 suppresses IL-1β in macrophages via the antioxidant regulator NRF2 (nuclear factor erythroid 2–related factor 2).^55^ In addition, REV-ERBα directly binds to the promoter region of Nlrp3 (NLR family pyrin domain containing 3), a major component of the inflammasome, thereby inhibiting its function in bone marrow–derived macrophages.^56–58^ Because macrophages are key sentinel cells of the cardiovascular system, any circadian oscillations will greatly affect their functions. This is particularly the case for the resident cells, as in this immune cell population, circadian oscillations have been described to generally exhibit larger amplitudes than other cells such as lymphocytes.

### Monocytes

Human total monocyte populations,^59^ as well as mouse inflammatory (CD115^+^Ly6C^high^)^60^ and noninflammatory monocytes (CD115^+^Ly6C^low^),^49^ exhibit circadian oscillations in their numbers in blood, peaking during the behavioral rest phase of humans and mice and exhibiting a trough during the active phase.^49^ This rhythm of Ly6C^high^ monocytes is disrupted on myeloid-cell–specific deletion of *Bmal1* (*Lyz2^cre^:Bmal1^flox^*).^60^ This deletion has been shown to affect the overall metabolism of the affected organism, leading to obesity.^60^

### Neutrophils

In steady-state conditions, both human and mouse neutrophil blood counts oscillate, with a peak during the midday and a trough at the start of the active phase.^61,62^ Moreover, the age composition of blood neutrophils exhibits a time-of-day difference. So-called aged neutrophils are characterized by morphological changes in their nucleus and increased maturation markers, such as Cxcr4 (chemokine CXC motif receptor 4) RNA levels and higher phagocytic capacity.^63^ In mice, the number of aged neutrophils in blood peaks at midday (ZT5), while young neutrophils predominate at ZT13 (fold change of ≈20 000 due to barely detectable levels at ZT13).^61^ This rhythmic neutrophil aging is regulated by *BMAL1* and leads to time-of-day differences in the antimicrobial activity in tissues.^64^ In humans, this aged neutrophil subset reaches its peak in the evening (7 pm, ≈2-fold),^65^ resulting in higher phagocytic activity in neutrophils isolated during the night.^65^ Although relatively few in numbers in murine blood, time-of-day–dependent infiltration of neutrophils to tissues has been suggested to alter overall tissue physiology.^66^ Given that neutrophils are the predominant immune cells found in human blood, any impact should, thus, be expected to be much greater in humans.

### Dendritic Cells

Lack of either *Nr1d1* or *Nr1d2* in bone marrow–derived DCs enhances the expression of maturation markers CD86 and MHCII (major histocompatibility complex II), along with proinflammatory cytokines such as IL (interleukin) 1β, IL6, and IL12b.^67^ The antigen processing capacity of DCs was found to be rhythmic with a peak during the rest phase of mice.^68^ CD80, another costimulatory molecule, is under direct circadian control of BMAL1 in DCs and governs circadian T-cell responses in mouse melanoma.^69^ DCs are critical immune cells, linking innate with adaptive immune responses. Therefore, circadian changes in their function have been linked with time-of-day dependency in vaccine efficacy and antitumor immunity.^51,69^

### T Cells

The mRNA levels of T-cell receptors and positive regulators of T-cell activation peak during the day, while negative regulators peak during the night.^70^ Conforming to these observations, in vitro studies demonstrated that T cells proliferate in a rhythmic manner and display oscillatory IFN-γ production.^71,72^ In vivo, mice vaccinated with OVA-loaded DCs during the day (ZT6)^70,71^ exhibit a significantly higher percentage of OVA-specific, IFN-γ producing effector T cells in the spleen, compared with those vaccinated during the night (ZT18, 1.5–5-fold).^70,71^ These data implicate T cells as important circadian effector cells.

### B Cells

In a mouse vaccination model, circadian variations observed in TLR9 (toll-like receptor 9) expression both at the mRNA and at the protein level in splenic B cells were shown to correlate with TLR9 responsiveness, which peaked at ZT19 (night).^73^ Other data have implicated humoral immune responses to be highly time-of-day dependent in both the preclinical and the clinical setting, thus linking circadian oscillations in B cells to vaccination efficacy.^74,75^

### Hematopoietic Stem and Progenitor Cells

Hematopoietic stem and progenitor cells (HSPCs) are also known to be subject to circadian regulatory patterns. HSPCs in the bone marrow generate various lineages of blood cells and many of their functions, such as proliferation, differentiation, or trafficking, exhibit time-dependent fluctuations that require tight coordination to ensure daily blood cell replenishment in both mice and humans.^76,77^ HSPCs are distinctive among adult stem cells due to their remarkable migratory capability even in adulthood.^78^ These cells move between the bone marrow and the bloodstream in response to chemotactic signals, with CXCL12 (C-X-C motif chemokine 12) being the most important among these signals. Similarly to leukocytes, HSPCs circulate in accordance with circadian oscillations, regulated by neural signals across various species, including humans.^79^ It has been shown that the postsynaptic neurotransmitter of the sympathetic nervous system noradrenaline exhibits a circadian rhythm in the murine bone marrow, peaking at night. This peak coincides with an increase in the number of cells in the G2/M and S phases of the cell cycle.^80^ Furthermore, noradrenaline was shown to directly stimulate the proliferation and migration of HSPCs in both mice and humans.^81,82^ Together, these findings underscore the profound influence of circadian rhythms on various progenitor and differentiated immune cell functions.

## CIRCADIAN RHYTHMS IN IMMUNE CELL-VASCULAR INTERACTIONS IN THE STEADY STATE

### Blood Vessels

Leukocytes are sentinels that continuously traffic through our bodies, shuttling between the vascular system and tissues. Within the vasculature, these immune cells can either circulate freely or interact with ECs through intricate interactions before infiltrating organs. Some organs, predominantly the lungs, even exhibit large intravascular pools of adherent leukocytes, which may not infiltrate the tissue but serve as reservoirs that can be quickly redirected to other sites after stimulation.^83^ Several studies have shown a correlation between elevated systemic blood leukocyte counts (which are strongly time-of-day dependent) and an increased risk of cardiovascular complications.^84^ Specifically, leukocyte recruitment to tissues and the interactions between adherent leukocytes and other components of the blood can worsen conditions, such as vaso-occlusive crises^66,85^ and lipopolysaccharide-induced lethality.^86^ Furthermore, recent evidence suggests that circadian leukocyte adhesion plays a critical role in the time-of-day–dependent onset of acute vascular inflammation.^11,60,81^ This implies that the timing of leukocyte adhesion in the steady state impacts the development of inflammation in blood vessels.

The leukocyte adhesion cascade is initiated when circulating leukocytes tether to ECs through highly glycosylated ligands such as PSGL-1 (P-selectin glycoprotein ligand-1) binding to selectins (P-/E-selectin) on the endothelium.^87,88^ This initial step brings the cells closer to the vessel wall and, at the same time, slows them down, allowing them to roll along the endothelium. Leukocytes can, thus, encounter various migratory factors on the EC surface, such as CXCL1 (an important chemokine for innate immune cell migration) or CCL21 (an important chemokine for adaptive immune cell migration). This engages chemokine receptors on leukocytes (eg, CXCR2 or CCR7, respectively), triggering G protein-coupled chemokine receptor signaling.^89^ Subsequently, integrins such as LFA-1 (lymphocyte function–associated antigen 1, also known as CD11a/CD18 or α_L_β_2_ integrin), macrophage antigen 1 (Mac-1, also known as CD11b/CD18 or α_M_β_2_ integrin), or VLA-4 (very late antigen 4, also known as CD49d/CD29 or α_4_β_1_ integrin) are activated and change their conformation to a higher affinity state. This enables leukocytes to interact with integrin counterreceptors on ECs, such as ICAM (intercellular adhesion molecule)-1 and ICAM-2 or VCAM-1 (vascular cell adhesion molecule 1), leading to firm adhesion of leukocytes to the endothelium. Once adherent, leukocytes crawl along the vessel wall and eventually emigrate from the vascular lumen into the surrounding tissue in a process known as transmigration.^19,88,90,91^

**Figure 1.**
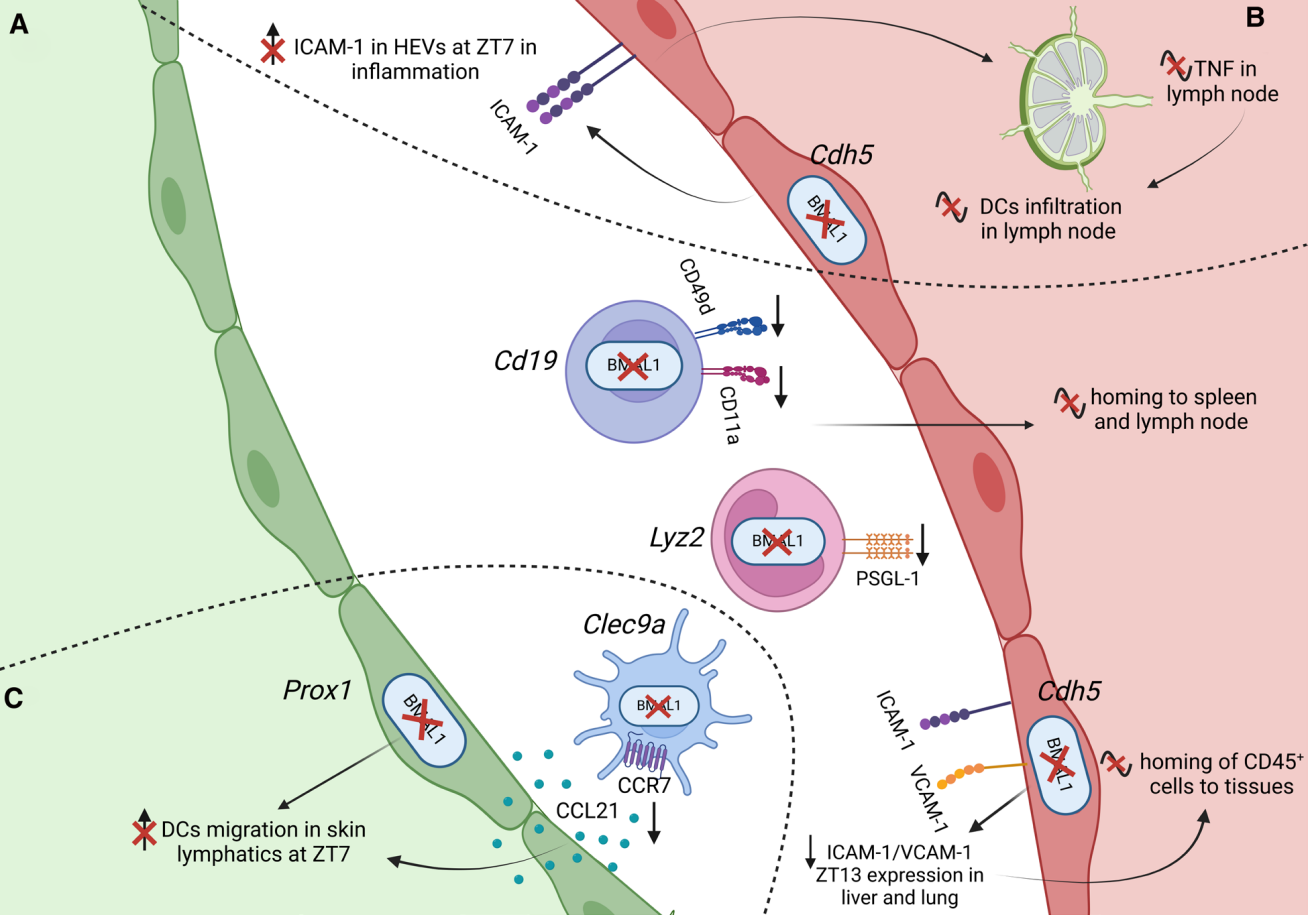
**Circadian rhythms control immune-vascular interactions at steady state.** Leukocytes emigrate from the blood to tissues in a rhythmic manner due to the oscillatory expression of adhesion molecules and chemokine receptors involved in the leukocyte adhesion cascade. This oscillatory expression is observed in both endothelial cells (ECs) and lymphatic ECs (LECs) of various organs, as well as in blood leukocyte subsets. **A**, Lineage-specific ablation of *Bmal1* in ECs (*Cdh5^creERT2^:Bmal1^flox^*) or leukocyte subsets such as B cells (*Cd19^cre^Bmal1^flox^*) and myeloid cells (*Lyz2^cre^:Bmal1^flox^*) leads to the abrogation of rhythmic of leukocyte migration to different organs. This rhythmic recruitment of leukocyte subsets to tissues is governed by promigratory factors on both ECs and leukocytes. **B**, Rhythmic expression of TNF (tumor necrosis factor) in the draining lymph node (LN) enhances BMAL1-controlled ICAM (intercellular adhesion molecule)-1 expression in high endothelial venules (HEVs) in an inflammatory scenario, resulting in increased dendritic cells (DCs) homing and lymphocyte infiltration to the draining LN. Lineage-specific ablation of *Bmal1* in ECs (*Cdh5^creERT2^:Bmal1^flox^*) abrogates these rhythms. **C**, Migration of mouse DCs into afferent lymphatic vessels of the skin follows a rhythmic pattern, peaking around Zeitgeber time (ZT) 7. This rhythmic migration is driven by the diurnal expression of adhesion molecules and chemokine-chemokine receptors on LECs and DCs. Lineage-specific ablation of *Bmal1* in conventional DCs (*Clec9a^cre^:Bmal1^flox^*), LECs (*Prox1^creERT2^:Bmal1^flox^*), or ECs (*Cdh5^creERT2^:Bmal1^flox^*) abrogates these rhythms.

Recent studies have highlighted the substantial influence of the circadian clock on immune cell distribution and locomotion. While leukocytes peak in the blood during an organism’s behavioral rest phase, they migrate into LNs and other tissues around the onset of the active phase.^49,50,92–94^ Genetic ablation of *Bmal1* abolishes the time-of-day difference in tissue infiltration, establishing a direct link between leukocyte trafficking behavior and the circadian clock^49,51,81,92,95^ (Figure 1; Table 1). Specifically, lack of *BMAL1* in ECs (using tamoxifen-inducible *Cdh5^creERT2^:Bmal1^flox^* mice) affects the circadian emigration pattern of most leukocyte subsets from blood.^49^ In addition, lineage-specific targeting of *Bmal1* in T cells (Cd4^cre^:*Bmal1*^flox^), B cells (Cd19^cre^:*Bmal1*^flox^), or myeloid cells (*Lyz2^cre^:Bmal1^flox^*) abrogates the steady-state circadian trafficking patterns of T and B cells to LNs, as well as B cells and neutrophils to the spleen, respectively. These effects seem to be mediated by an abrogation of oscillatory migratory factors on both ECs and leukocytes,^49^ indicating oscillations in leukocyte and EC receptor-ligand pairs to be of equal importance (Figure 1A; Table 1). ECs have been the best studied in this aspect. A direct influence of the circadian clock machinery on the expression of the *Icam1* gene in ECs has been demonstrated. In an in vitro EC culture model, the exogenous expression of the circadian gene *Clock* was found to upregulate *Icam1* transcriptional activity and expression (≈2-fold).^96^ ChIP (chromatin immunoprecipitation) analyses further revealed that *Clock* binds to an E-box motif in the enhancer region of the *Icam1* gene. Serum shock treatment of ECs, a method used to assess circadian oscillations of cultured cells in vitro, showed an oscillatory *Icam1* gene expression, indicating that *Icam1* may indeed be a directly *Clock*-controlled gene. Furthermore, *Clock* overexpression in ECs promoted the adhesion of mononuclear cells to ECs via ICAM-1^96^ (Table 1). In addition to *Clock*, ChIP assays of LNs harvested from wild-type mice on skin painting with the irritant FITC (fluorescein isothiocyanate), demonstrated BMAL1 to directly bind to E-box regions within the *Icam1* promoter. This interaction exhibited rhythmic occupancy, being higher after stimulation with FITC during the day than at night.^51^ *Icam1* induction was further dependent on the rhythmic expression of the inflammatory cytokine TNF (tumor necrosis factor) in the LN, indicating that the circadian clock and inflammation coregulate ICAM-1 expression. Endothelial lack of *Bmal1* (*Cdh5^creERT2^:Bmal1^flox^*) abrogated time-of-day differences in ICAM-1 expression in ECs, including high endothelial venules, the sites of leukocyte infiltration into the LN from blood^49,51^ (Figure 1B; Table 1). Together, these data highlight *Icam1* as an important circadian-controlled gene in the leukocyte adhesion cascade. On leukocytes, rhythmic expression of migratory factors has been described in mice in PSGL-1, CD11a (α_L_ integrin), CD49d (α_4_-integrin), L-selectin, and the chemokine receptors CCR7 and Cxcr4.^49,92^ *Bmal1* deficiency in specific leukocyte lineages has been shown to abrogate oscillations in CD11a and CD49d, as well as CCR7 and CXCR5 in B and T cells and PSGL-1 surface expression in neutrophils, respectively^49,92^ (Figure 1A; Table 1). In humans, CD11a, ICAM-1, L-selectin, and Cxcr4 have been shown to display diurnal fluctuations in leukocytes.^97^ Together, ample evidence shows that interactions between leukocytes and blood ECs are highly rhythmic in the steady state due to a direct effect of the circadian clock machinery on the rhythmic expression of migratory factors. These physiological oscillations define the location and phenotype of immune cells across the day and are, thus, critical in defining the response to injury or pathological insult at any given time.

**Table 1.**
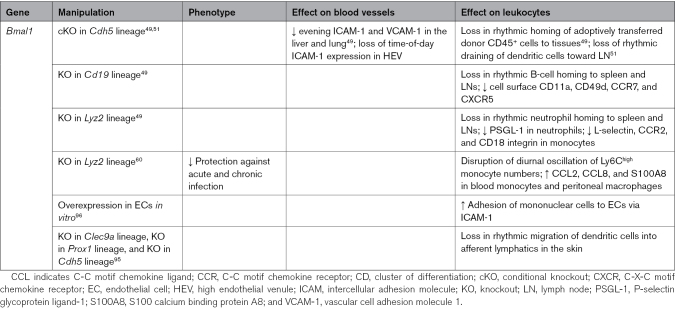
Circadian Immune-Vascular Interactions in Steady State

Although steady-state leukocyte recruitment to tissues occurs almost exclusively from small-caliber veins such as postcapillary venules, inflammation also induces leukocyte adhesion to arterioles and arteries.^98^ In a mouse model of acute inflammation, induced via the local administration of TNF, genes involved in the trafficking cascade, such as *Icam1*, *Vcam1*, *Sele* or *Selp* (encoding for E- and P-selectin, respectively), *Cxcl1*, *Cxcl2*, and *Ccl2*, were shown to exhibit rhythmic transcription across the day in mouse ECs.^99^ These rhythmic expression patterns lead to variations in leukocyte adhesion to arteries and veins, with peaks in adhesion to arteries in the morning (≈1.5-fold) and to veins at night (≈4-fold).^99^ Overall, these data highlight that the interactions between immune cells and blood vessels are strongly governed by the circadian clock machinery both at steady state and in inflammation.

### Lymphatic Vessels

Similar to circadian leukocyte infiltration into LNs from the blood, cells also drain from tissues in a rhythmic manner to LNs, via afferent lymphatics. Migration of mouse DCs into afferent lymphatic vessels of the skin follows a circadian pattern, peaking around ZT7 and troughing at ZT19 (≈3-fold).^95^ This rhythmic migration is driven by the diurnal expression of several adhesion molecules on lymphatic ECs, including CCL21, LYVE-1 (lymphatic vessel endothelial hyaluronan receptor 1), CD99, and JAM-A (junctional adhesion molecule A), in addition to DC-expressed CCR7. This has been substantiated by lineage-specific ablation of *Bmal1* in lymphatic ECs (*Prox1^creERT2^:Bmal1^flox^*), ECs (*Cdh5^creERT2^:Bmal1^flox^*), or conventional DCs (*Clec9a^cre^:Bmal1^flox^*), resulting in a loss of oscillations in these parameters (Figure 1C; Table 1). ChIP analyses further demonstrated that BMAL1 can directly control the expression of CCL21, CCR7, and LYVE-1, through its rhythmic binding to their promoter regions, which peaks at ZT13.^95^ CCL21 expression has also been demonstrated to exhibit diurnal oscillations in human skin samples, with a peak in the morning (8:00 hours), indicating that this trafficking phenotype might extend to humans. Rhythmic DC trafficking continues from the afferent lymphatics into the draining LN. In mice, DCs infiltrate LNs in greater numbers (≈2-fold) when an inflammatory reaction in the skin was induced by FITC painting at ZT7 compared with animals painted at ZT19.^51,95^ This resulted in a time-of-day–dependent increase in LN cellularity in this inflammatory model and ultimately in rhythmic adaptive immune responses^51^ (Figure 1B; Table 1). These oscillations ensure a higher probability of T cells interacting with DCs, leading to stronger adaptive immune responses when both cell types are found at higher numbers in the LNs.

In addition to rhythmic infiltration of leukocytes from both blood and afferent lymphatics, lymphocyte egress from the LN also occurs rhythmically, via efferent lymphatics. This is controlled by the circadian expression of the egress factor S1pr1 (sphingosine-1-phosphate receptor) in lymphocytes. S1pr1 is a pivotal molecule in the regulation of LN egress and displays robust diurnal oscillations in these cells, peaking in the early afternoon (between ZT5-7), when egress is maximal, and showing a ≈3-fold increase compared with ZT19-21.^92^ Studies targeting *S1rp1* genetically in lymphocytes using *Cd4^cre^:S1pr1^flox/+^* heterozygous mice suggest that the oscillatory expression of *S1pr1* controls rhythmic lymphocyte egress.^92^ Together, these findings underscore a highly regulated interplay between immune cells and ECs in both blood and lymphatic vascular beds, which is orchestrated by circadian clocks and strongly influences the leukocyte infiltration to and emigration from tissues.

## CIRCADIAN RHYTHMS IN IMMUNE CELL-VASCULAR INTERACTIONS IN DISEASE

### Atherosclerosis

Atherosclerosis is a chronic inflammatory vascular disease, characterized by plaques containing lipids, cells, and cellular debris. It is estimated to affect about a quarter of the population worldwide^100^ and presents a great risk for acute cardiovascular complications, such as MI and stroke. The development of atherosclerotic plaques is a long process involving complex mechanisms.^101^ In this section, we discuss the implication of circadian rhythms on leukocyte-EC interactions in atherosclerosis.

**Figure 2.**
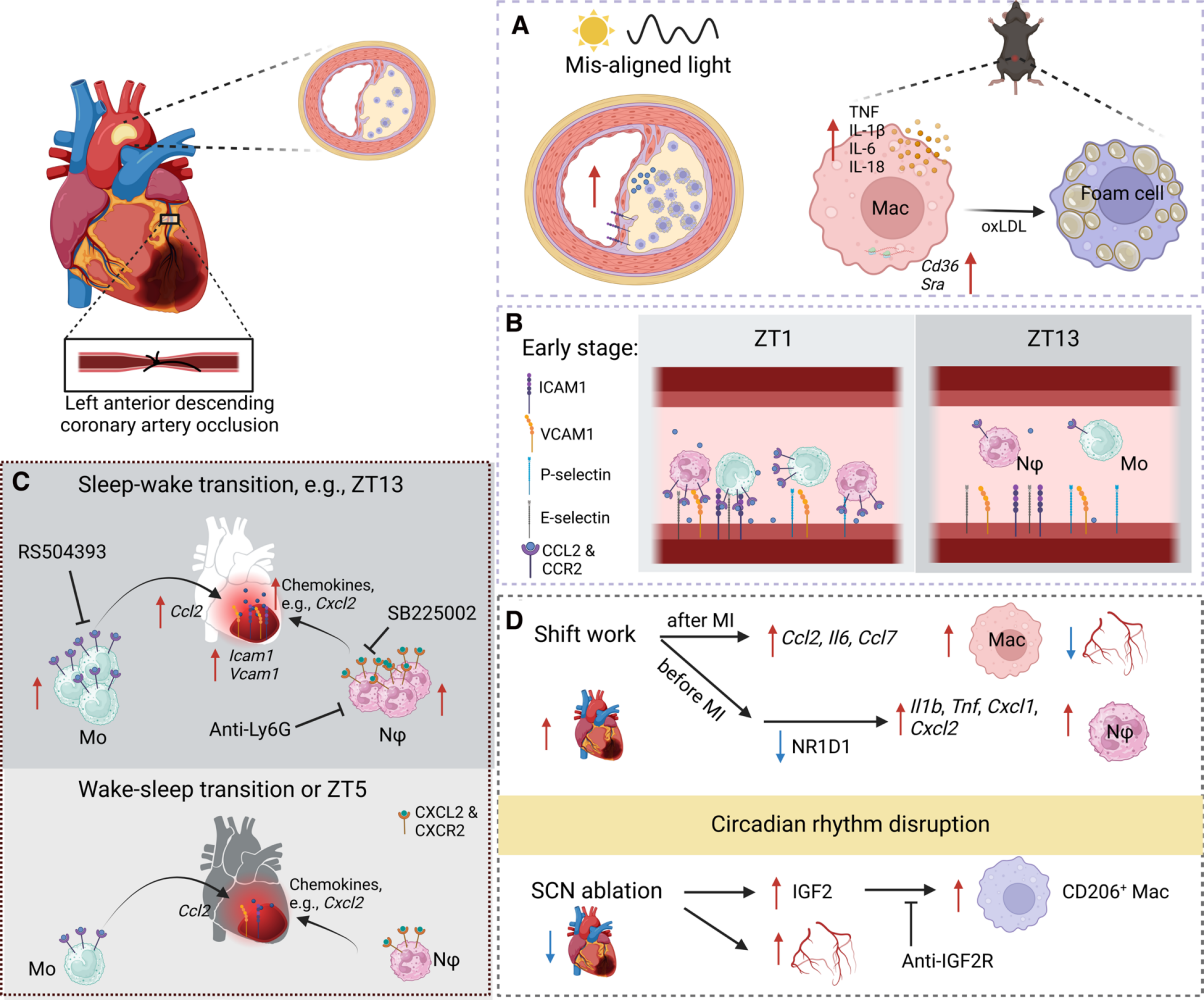
**Circadian rhythms control immune-vascular interactions in atherosclerosis and myocardial infarction (MI).** Monocytes (Mo) and neutrophils (Nφ) adhere to arteries and infiltrate into the heart in a time-of-day–dependent manner, which dictates the circadian outcome of atherosclerosis and MI. Disruption of circadian rhythms generally exacerbates the disease phenotype. **A**, Circadian rhythm disruption, such as by misaligned light, increases atherosclerotic lesions and elevates their *Icam1* and CCL2 levels. Peritoneal macrophages (Mac) from circadian-disrupted mice are more inflammatory and more likely to form foam cells in response to oxLDL (oxidized low-density lipoprotein). **B**, In the early stages of atherosclerosis, arteries show higher levels of CCL2 on the endothelial surface at Zeitgeber time (ZT) 1 compared with ZT13, leading to greater monocyte and neutrophil adhesion at ZT1. **C**, In MI models induced by left anterior descending coronary artery occlusion, MI damage is more severe at ZT13 compared with ZT5 or wake-sleep transition. Higher levels of adhesion molecules and chemokines are present at ZT13, leading to increased monocyte and neutrophil infiltration to the heart. The blockade of this infiltration by the CCR2 inhibitor RS504393, the CXCR2 inhibitor SB225002, or the depletion of neutrophils by anti-Ly6G antibodies protects the heart from severe MI damage at ZT13. **D**, Circadian rhythm disruption by shift work or by surgical lesion of the suprachiasmatic nucleus (SCN) has the opposite effects on MI damage. Post-MI shift work increases cardiac inflammation and macrophage infiltration and suppresses new coronary blood vessel formation and associated infarct healing. Prior-MI shift work increases cardiac inflammation and neutrophil infiltration by suppressing *Nr1d1* expression. However, SCN lesion promotes blood vessel recovery and associated blood flow and cardiac anti-inflammatory CD206^+^ macrophage infiltration, which is mediated by IGF2 (insulin-like growth factor 2) and could be suppressed by an anti-IGF2R antibody. CCL2 indicates C-C motif chemokine ligand 2; CCR2, C-C motif chemokine receptor 2; CXCR2, C-X-C motif chemokine receptor 2; ICAM1, intercellular adhesion molecule 1; IGF2R, insulin-like growth factor 2 receptor; and IL, interleukin.

Disrupted circadian rhythms are a risk factor for atherosclerosis. In night shift workers, the carotid intima-media thickness measured by ultrasound was found to be increased and the likelihood of having carotid plaques to be 1.5- to 2-fold higher than in nonshift workers.^102–104^ Similar effects have been observed in mouse models with disrupted light:dark phases. Hyperlipidemic APOE^*3-Leiden.CETP^ mice (a mouse model of atherosclerosis with mutations in apolipoprotein E) housed in light-dark cycles with weekly 6-hour light phase advances or delays or in cycles with weekly alternating light-dark cycles (12-hour shifts) for 15 weeks displayed increased risks of developing atherosclerosis, with respect to both lesion size and percentage of severe lesions with a lipid core that also contains thick layers of fibrous connective tissue (up to 2-fold) compared with mice housed in regular light-dark cycles.^105^ In contrast, the same mouse strain exposed to constant light for 14 weeks did not show an increased incidence of atherosclerosis, indicating potential differential effects of continuous versus misaligned light exposure on atherosclerotic plaque burden.^106^ In line with these data, increased atherosclerosis lesions have been observed under misaligned light conditions in low-density lipoprotein receptor knockout (*Ldlr*^−/−^) mice^107^ and apolipoprotein E knockout (*Apoe*^−/−^) mice.^108^ Again, constant light conditions did not worsen the already enhanced disease burden observed in female *Apoe*^−/−^ mice.^109^ Notably, higher levels of *Icam1* and CCL2 (1.5–2-fold), along with increased levels of macrophages (≈2-fold), were observed in the intermediate atherosclerotic lesions in weekly 12-hour shifted mice compared with control mice.^105^ Moreover, peritoneal macrophages harvested from circadian rhythm-disrupted mice secreted higher levels of inflammatory cytokines (TNF, IL-1β, IL-6, and IL-18), expressed higher levels of cholesterol transporter genes (*Cd36* and *Sra*), and displayed a greater propensity (≈1.6-fold) to form foam cells in the presence of oxidized LDL^110^ (Figure 2A). Collectively, this indicates that disruption of circadian rhythms promotes inflammation and macrophage levels in the atherosclerotic lesions and worsens the disease burden.

Monocytes, the precursor of macrophages in atherosclerotic lesions, and neutrophils adhere to the carotid artery and aorta in an oscillatory manner in the early stages (4 weeks of high-fat diet) of atherosclerotic *Apoe*^−/−^ and *Ldr*^−/−^ mice.^111^ Adhesion peaks at ZT1 and shows a trough at ZT13 (1.5–3-fold). This is in phase with the peaks and troughs of monocyte and neutrophil counts in peripheral blood and aligns with other models of inflammation-induced oscillations in leukocyte adhesion to arteries and arterioles.^99,111^ Importantly, however, this pattern is inverse to rhythms in leukocyte adhesion observed in veins and venules, which peak in the evening and trough during the day. Mechanistically, levels of the chemokine CCL2 were demonstrated to be higher on arteries at ZT1 (≈1.6-fold), and the expression of CCR2 and CCR5 was higher (≈1.4-fold) on monocytes and neutrophils at ZT1 compared with ZT13 in this model^111^ (Figure 2B). Oscillatory expression of CCL2 was shown to be primarily derived from monocytes and neutrophils. In addition, rhythmic plasma CCL2 levels were abolished in myeloid *Bmal1* deficient mice (*Lyz2^cre^:Bmal1^flox^*). Blockade of the CCL2-CCR2 signaling axis via the CCR2 antagonist RS102895 and the use of *Ccr2^−/−^* mice effectively abolished rhythmic adhesion of monocytes and neutrophils to carotid arteries.^111^ Of therapeutic relevance, administration of RS102895 (a drug that exhibits a half-life of <1 hour) to atherosclerotic *Apoe^−/−^* mice 8 hours before the peak of morning monocyte adhesion significantly suppressed (≈2-fold) the formation of atherosclerotic lesions while leaving monocyte adhesion to the microcirculation (ie, venules, which peaks in the evening^99^) intact.^111^ This indicated that chronotherapeutic blockade of CCR2 might improve the efficacy of atherosclerosis treatments while leaving venular trafficking routes intact, important for immune surveillance. Further investigations are, thus, warranted to explore its potential application in the treatment of late-stage atherosclerotic disease in humans.

The impact of circadian rhythms on atherosclerosis has been further demonstrated by genetic manipulation in mouse models (Table 2). *Ldlr*^−/−^ mice and *Apoe*^−/−^ mice with an additional dominant-negative mutant of the gene *Clock* (*Clock^Δ19^*) exhibit enhanced levels of atherosclerosis compared with *Ldlr*^−/−^ (1.6–4-fold) and *Apoe*^−/−^ (22–34-fold) animals, respectively.^117^ Besides higher plasma lipid levels, higher inflammatory cytokines, including IL-12 and G-CSF (granulocyte colony-stimulating factor), were found in the plasma of *Clock^Δ19^ Apoe^−/−^* mice compared with *Apoe*^−/−^ mice alone. In addition, *Clock^Δ19^Apoe^−/−^* animals exhibit enriched numbers of macrophages in their atherosclerotic lesions. Using bone marrow–derived macrophages as an in vitro model, elevated levels of inflammation (*IL12*, *IL6*, *Tnf*, and *Csf3*), increased lipid uptake (CD36 and SR-A1), and reduced cholesterol efflux (ABCA1 [ATP binding cassette subfamily A1] and ABCG1 [ATP binding cassette subfamily G1]) were detected in bone marrow–derived macrophages generated from *Clock^Δ19^Apoe^−/−^* mice^117^ (Table 2). This regulation of cholesterol efflux was specific to the circadian gene *Clock* and involves the transcription factor USF2, as only *Clock* knockdown but not the knockdown of *Per1*, *Cry1*, or *Bmal1* in bone marrow–derived macrophages led to a reduction in *Abca1* mRNA levels.^117^ Given the pivotal role of macrophages in atherosclerosis formation, it was found that *Apoe*^−/−^ mice receiving bone marrow from *Clock^Δ19^Apoe^−/−^* mice also exhibited more severe atherosclerosis (2–2.7-fold) than those receiving bone marrow from *Apoe*^−/−^ mice. This suggests that macrophage dysfunction is a key factor in the aggravated atherosclerosis phenotype observed in *Clock^Δ19^* mice,^117^ and altered clock function in macrophages may contribute to atherosclerosis in patients.

**Table 2.**
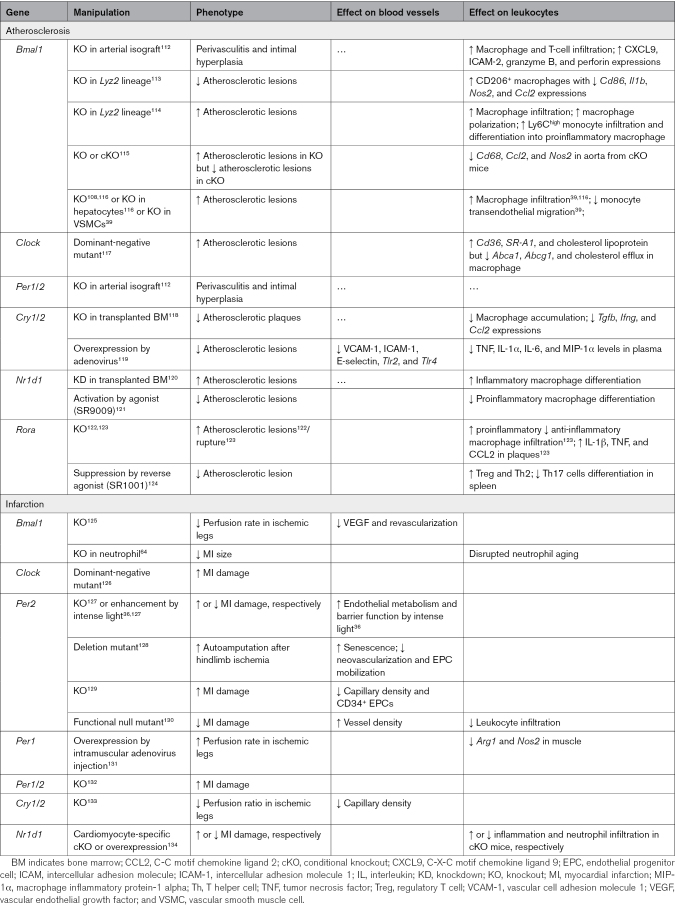
Circadian Immune-Vascular Interactions in Atherosclerosis and Infarction

In accordance with the *Clock* mutant data discussed above, global deficiency of *BMAL1* as seen in double-deficient *Apoe*^−/−^*Bmal1*^−/−^ and *Ldr*^−/−^*Bmal1*^−/−^ mice accelerated atherosclerosis (2–7-fold) and promoted macrophage infiltration (≈4-fold) in lesions.^116^ Similarly, hepatocyte specific *Bmal1* (*Alb^Cre^:Bmal1^flox^*) deficiency in *Apoe*^−/−^ mice^116^ and vascular SMC-specific *Bmal1* (*Smmhc^creERT2^:Bmal1^flox^*) deficiency in western diet-fed mice^39^ also promoted atherosclerosis (≈2-fold) and monocyte transendothelial migration^39^ (Table 2). However, when *Bmal1* is deleted in the myeloid lineage (*Lyz2^cre^:Bmal1^flox^*), contradictory findings have been reported.^113,114^ In the *Apoe^−/−^* background, *Bmal1* deletion in myeloid cells increased the size of atherosclerotic lesions, as well as the numbers of macrophages and Ly6C^high^ cells, and skewed macrophage polarization toward a rather proinflammatory phenotype.^114^ When Ly6C^high^ monocytes were adoptively transferred and tracked, it was observed that *Bmal1* deficiency prompted increased trafficking of Ly6C^high^ monocytes to atherosclerotic lesions, preferential differentiation of Ly6C^high^ monocytes into proinflammatory macrophages, and, subsequently, an augmentation in macrophage content and lesion size in the carotid arteries.^114^ In contrast, *Bmal1* deletion in myeloid cells yielded a strikingly different phenotype in the *Ldr*^−/−^ background. Here, the result was a substantial reduction in lesion burden (≈2-fold), with plasma glucose and lipid levels remaining comparable to those of *Ldr*^−/−^ mice.^113^ Furthermore, there were no significant changes in CD11b^+^ myeloid cell levels in the lesions. Instead, an increase in CD206^+^ anti-inflammatory macrophages was observed, coupled with a decrease in expression of *Cd86*, *Il1b*, *Nos2*, and *Ccl2* in the aorta^113^ (Table 2). Further investigations are, thus, required to understand the observed differences in *Bmal1* deficiency in these different atherosclerotic-prone genetic backgrounds. Whether *Bmal1* deletion is constitutive or inducible adds another layer of complexity to the outcome of atherosclerosis. When *Bmal1* is constitutively deleted in the *Ldr*^−/−^ background, atherosclerosis is enhanced (≈2.5-fold).^115^ However, when *Bmal1* deletion in various tissues is induced by tamoxifen (*Esr^creERT2^:Bmal1^flox^*) in adult *Ldr*^−/−^ mice, the severity of atherosclerosis and the expression of *Cd68*, *Ccl2*, and *Nos2* in the aorta are reduced (2–4-fold)^115^ (Table 2). The merit of the inducible *Bmal1* deletion model is that developmental functions of BMAL1 are not affected so that mice retain normal rhythmic locomotor activities before the tamoxifen treatment.^115^ Thus, in the constitutive *Bmal1* deletion atherosclerosis model, any alterations caused by *Bmal1* deletion during development remain unassessed.

The circadian rhythm of blood vessels themselves also plays a role in atherosclerosis. In an artery transplant model, perivasculitis and intimal hyperplasia—indicative of transplant arteriosclerosis—only occur in arterial isografts from *Bmal1*^−/−^ or *Per1*^−/−^*Per2*^−/−^ double-deficient donors but not in arterial isografts from wild-type donors, regardless of whether the recipients were wild-type or *Bmal1*-deficient.^112^ Inflammation is apparent within the *Bmal1*-deficient grafts in wild-type mice, as evidenced by increased macrophage and T-cell infiltration and higher expression levels of *Cxcl9*, *Icam2*, *Gzmb*, and *Perforin* (1.6–3.3-fold)^112^ (Table 2). However, B- and T-cell infiltration is dispensable for the inflammatory phenotype because transplant arteriosclerosis persists in *Bmal1*-deficient aortic grafts transplanted into recipients deficient in the adaptive immunity recombination-activating gene (RAG-1).^112^ These data demonstrate a remarkable and apparently intrinsic vascular function of circadian clocks to condition transplant arteriosclerosis. This may need to be taken into account when performing vascular transplants in the clinic.

Besides *Bmal1* and *Clock*, also the clock genes *Nr1d1*, *Cry1/2*, and *Rora*, have been implicated in atherosclerosis (Table 2). *Nr1d1* knockdown^120^ or treatment with the REV-ERBα agonist SR9009^121^ accelerated (≈1.5-fold) or suppressed atherosclerosis and reduced the acquisition of a proinflammatory phenotype in macrophages, respectively. Yet, a potential effect on atherosclerosis by *Cry1/2* and *Rora* has been controversial. For these genes, atherosclerosis and atherosclerosis-related inflammation were mitigated through different experimental approaches. These included bone marrow transplants from *Cry1^−/−^Cry2^−/−^* double-deficient mice^118^ and the overexpression of CRY1 via adenovirus infusion.^119^ While these opposite interventions surprisingly had similar suppressive effects, the specific target genes differed slightly, and CRY1 overexpression was assessed only in the aorta. *Rora*^−/−^ mice show enhanced levels of atherosclerosis (6–7.5-fold),^122^ atherosclerotic plaque rupture (≈2-fold),^123^ polarization, and numbers of inflammatory macrophage^123^ in atherosclerotic models. However, the retinoic acid receptor–related orphan receptor α/γ inhibitor SR1001 reduced atherosclerosis (≈2-fold), promoted Treg (regulatory T cell) and Th2 differentiation, and suppressed Th17 cell differentiation in the spleen.^124^ These varying outcomes may be attributed to the different genes and cell types that were targeted in each study. These data firmly link dysregulated circadian vascular function with cardiovascular disease. Further research is needed to determine the mechanism of atherosclerosis regulation by circadian clock genes, with special attention to the target gene and cell type, as well as the use of constitutive versus inducible gene knockouts.

### Infarction

Infarction refers to tissue necrosis due to insufficient blood supply. Typical examples of infarction include MI and stroke, which are the main killers worldwide^135^ and are often caused by the rupture of atherosclerotic plaques. It has long been observed that MI,^136^ sudden cardiac death,^137^ and stroke^138^ occur more frequently in the morning in humans, indicating a circadian regulation. Many factors contribute to the variation of infarction across the day,^23^ including the higher activity of platelets in the morning and the rise in blood pressure at this time.^139^ Furthermore, platelets interact more with leukocytes in the artery at ZT1 (morning) compared with ZT17 (evening) and are more likely to form an induced thrombus in arteries in the morning.^99^ In contrast, thrombi are more likely to form in veins in the afternoon^99^ or evening^81^ due to a phase-shift in the peak of leukocyte-platelet^99^ and leukocyte-erythrocyte^81^ interaction. These data demonstrate that cardiovascular disease is strongly linked to circadian (dys)-functional interactions between the vascular wall and cells within the blood.

The severity of infarction damage is time-of-day dependent. In patients with MI, an association exists between the time-of-day of symptom onset and the infarction size or survival.^140–147^ In mice, a higher severity is generally observed when infarction occurs at the day-night transition in mice.^35,64,132,148–154^ The main mouse MI model is generated by temporary or permanent ligation of the left anterior descending (LAD) coronary artery. In one set of experiments, mice were phase-shifted to different time zones and surgeons were blinded to the time of mice, limiting the variable to the time zone of the mouse and not the surgeon. MI size after 45 minutes of ischemia followed by 60 minutes of reperfusion was 78% larger in the group of mice where MI was performed at ZT10-12 than in the ZT0-2 group.^148^ This study also identified that RCAN1 (regulator of calcineurin 1) mediated the time-of-day changes in the susceptibility of the heart to ischemia-reperfusion damage and that FK506, an inhibitor of calcineurin, decreased the infarct size induced at ZT10-12 times. Notably, calcineurin is involved in immune activation and FK506 is commonly used as an immunosuppressant, indicating that the time-of-day damage may be caused by leukocytes. Indeed, neutrophil infiltration to the murine heart shows a peak at ZT13 and a nadir at ZT5 at the steady state (≈2.5-fold).^35^ This peak overlaps with the peak in cardiac expression of chemokines (*Cxcl1*, *Cxcl2*, *Cxcl5*, *Ccl3*, and *Ccl5*) and adhesion molecules (*Icam1* and *Vcam1*), as well as neutrophil expression of *Cxcr2*. Consequently, MI as induced by permanent LAD ligation is more severe at ZT13 compared with ZT5.^35,132^ The increase in neutrophil infiltration to the heart after MI at ZT13 compared with ZT5 was accompanied by a decrease in neutrophil counts (3–4-fold) and an increase in granulocyte–monocyte progenitors (≈2-fold) in the bone marrow. Furthermore, treatment with the CXCR2 antagonist SB225002 reduced the infiltration of CXCR2^+^ neutrophils to the heart after MI (≈2-fold) and neutrophil depletion with an anti-Ly6G antibody improved the recovery after MI at ZT13^35^ (Figure 2C). Besides neutrophils, Ly6C^high^ monocytes also show a circadian infiltration to the heart at steady state (≈3-fold), with a peak at ZT13 and a trough at ZT5, which coincides with the peak *Ccl2* expression at ZT13 in cardiac tissues. Concomitantly, MI at ZT13 leads to higher numbers of Ly6C^+^ monocytes and macrophages in the heart (1.6–2-fold), which could be abrogated with the CCR2 inhibitor RS504393^150^ (Figure 2C). However, whether there is a causal relationship between time-of-day–dependent monocyte infiltration and MI remains yet to be determined. These studies firmly link the circadian infiltration of myeloid cells to MI-associated damage.

Disruption of circadian rhythms, such as induced by shift work, has a harmful impact on the outcome of infarction in patients.^133,134^ Shift work is associated with an increased incidence of MI.^155^ In addition, shift work increases MI injury in humans and mice.^134^ In a clinical cohort composed of 186 patients, shift work was shown to be associated with increased cardiac infarct size as measured by magnetic resonance imaging. During a median follow-up of 5 years, shift work was shown to be associated with increased risks of major adverse cardiac events (adjusted hazard ratio, 1.92).^134^ In a shift work mouse model where an 8-hour phase advance was introduced twice per week for 8 weeks, phase shifting exacerbated the ZT12 time point of MI reperfusion damage (30-minute occlusion of LAD). Moreover, shift work as induced by reversing the photoperiod twice per week for 12 weeks also significantly promoted MI reperfusion injury (60-minute occlusion of LAD) in sheep.^134^ Mechanistically, decreased RNA and protein levels of *NR1D1* (ie, REV-ERBα) were observed in human, mouse, and sheep hearts in MI that occurred after shift work (Figure 2D), while other circadian genes remained unchanged or increased.^134^ Conditional cardiomyocyte-specific *Nr1d1* knockout mice (*Myh6^CreMer^:Nr1d1^flox^* and *Nr1d1*^cKO^) showed worsened cardiac damage after MI. This indicated *Nr1d1* to be a critical circadian gene in the process. Indeed, TNF was shown to suppress *Nr1d1* expression in primary mouse cardiomyocytes, and RNA-sequencing analyses revealed an enrichment of inflammatory pathways in *Nr1d1*^cKO^ mice compared with controls. Furthermore, neutrophil numbers and levels of *Il1b*, *Tnf*, *Cxcl1*, and *Cxcl2* were elevated in *Nr1d1*^cKO^ MI hearts.^134^ Conversely, cardiac-specific REV-ERBα expression by intramyocardial injection of an AAV (adenoviral vector) encoding *Nr1d1* (AAV-Nr1d1) under a cardiomyocyte-specific cTnT (cardiac troponin-T) promoter or REV-ERBα activation by intraperitoneal injection of the agonist SR9009 both alleviated MI damage, regardless of shift work, in both mice and sheep. Neutrophil infiltration and inflammatory cytokine expression were suppressed in both models in mice.^134^ This demonstrates that shift work can promote MI damage by suppressing *Nr1d1*, which reduces inflammation and protects the heart from leukocyte-induced MI damage (Table 2).

Not only prior disruption of circadian rhythm promotes MI damage but also short-term disruption of circadian rhythms post-MI leads to increased long-term MI damage.^156^ Mice with a permanent LAD ligation performed at ZT1-4 were maintained for 5 days after injury either in a normal 12-hour light:12-hour dark cycle or in a cycle that consisted of a 10-hour light:10-hour dark schedule. Strikingly, mice with short-term diurnal disruptions after MI displayed worse heart functions 8 weeks after MI compared with mice without diurnal disruptions after MI.^156^ Short-term diurnal disruption post-MI also increased cardiac inflammatory cytokine *Il6*, *Ccl2*, and *Ccl7* expressions 36 hours after MI and increased cardiac macrophage infiltration 3 and 7 days after MI. This was associated with reduced blood vessel formation in the infarct region of diurnal disrupted mice 1 week after MI, which is associated with a worse recovery^156^ (Figure 2D). This indicates that altered light schedules and potentially affected daily routines might be important to adjust also in patients recovering from MI.

In contrast to shift work, disruption of circadian rhythms by surgical lesion of the SCN attenuates MI damage^132^ (Figure 2D). In a double-blind experiment, SCN lesions attenuated (≈2-fold) cardiac damage and cardiac fibrosis 4 weeks after MI compared with sham control animals. Additionally, an SCN lesion was associated with an increase (2–3-fold) in the number of ECs in the border zone between MI and healthy tissue in mice. Circadian desynchronization by constant light had similar effects on MI as SCN lesions in mice.^132^ This indicates that a complete lack of circadian oscillations may result in a milder phenotype than an alteration of oscillations, the latter of which may be associated with a higher stress burden. However, why these forms of circadian rhythm disruption (SCN lesion and constant light) result in different effects on MI from shift work remains to be further studied. Nevertheless, RNA sequencing of early MI heart tissues revealed a remarkable difference in immune pathways between sham-operated and SCN-lesioned samples on day 3. A greater number of immune cells, particularly of CD206^+^ anti-inflammatory macrophages (≈1.4-fold), were observed in mouse hearts from SCN-lesioned mice compared with sham control mice.^132^ This SCN lesion–mediated protection from MI-induced cardiac impairment is surprising and probably dependent on neurohumoral factors, as the same study found *Per1^−/−^Per2^−/−^* double-deficient mice to exhibit more deterioration of pathophysiologic function and cardiac fibrosis after MI.^132^ Mechanistically, the authors demonstrated that this may be due to an increased (≈2-fold) IGF2 (insulin-like growth factor 2) concentration in the serum of SCN-lesioned mice that promoted the differentiation of CD206^+^ macrophages because anti-IGF2R (anti-insulin-like growth factor 2 receptor) antibodies abrogated the protective effect of SCN lesion on MI.^132^ Together, these data indicate that the SCN function is directly linked to MI outcome.

The circadian control of MI has been intensively studied in *Per2*-deficient mouse models (Table 2). Despite some controversy,^130^ *Per2* deficiency appears to primarily exacerbate the damage associated with infarction in mice.^36,127–129^ Furthermore, ischemic preconditioning (4 cycles of 5-minute ischemia, 5-minute reperfusion)–mediated cardiac protection was also abrogated in *Per2*^−/−^ mice. In adenosine receptor–mediated myocardial adaptation to ischemia or hypoxia, *Per2* expression was found to be elevated (3–6-fold) in both mice and humans^36,127^ due to both transcriptional and translational mechanisms, and posttranslational suppression of proteasomal degradation.^127^ Moreover, stabilization of *Per2* in the heart by exposing mice to 4 hours of intense light (13 000 versus 200 lux) resulted in transcriptional induction of glycolytic enzymes and *Per2*-dependent cardioprotection from ischemia.^127^ Another study confirmed the protective effects of intense light on MI.^36^ As might be expected, this enhancement and cardioprotection by intense light were dependent on an intact vision of mice. Mechanistically, intense light was shown to protect the heart from ischemia-reperfusion injury in an EC-*Per2*–dependent manner, as the positive effect was abolished in endothelial-specific *Per2*^−/−^ (*Cdh5^cre^:Per2^flox^*) mice. Intense light enhanced the metabolism and maintained the barrier function of the endothelium via increased HIF1A (hypoxia-inducible factor 1-alpha) transcription.^36^ These protective effects of *Per2* on infarction and ECs have also been demonstrated in hindlimb ischemia models.^128,129^ Here, *Per2* mutation or deficiency promoted EC senescence^128^ and suppressed neovascularization by inhibiting endothelial progenitor cell mobilization.^128,129^ Together, these data suggest that light modulates heart function and may provide possible therapeutic avenues in the future. However, how *Per2* deficiency affects the immune system and its contribution to infarction damage is largely unexplored. Furthermore, how light signals are relayed to ECs in this scenario remains to be elucidated.

Other circadian clock genes are also involved in regulating infarction damage (Table 2). Intramuscular injection of an adenovirus driving *Per1* expression was shown to increase the perfusion ratio of ischemic legs in mice up to 2-fold. *Per1* overexpression increased the expression of *Arg1* and decreased the expression of *Nos2*, which are often used as markers defining anti-inflammatory and inflammatory macrophages, respectively.^131^ *Bmal1*^−/−^ mice^125^ and *Cry1^−/−^Cry2^−/−^* double-deficient mice^133^ exhibited worse recovery and angiogenesis after hindlimb ischemia. Also, *Clock^∆19/∆19^* mice showed a worse outcome after MI.^126^ However, neutrophil-specific *Bmal1*–deficient mice (*hMRP8^CRE^:Bmal1^flox^*) had smaller infarct sizes (≈2-fold) and slightly higher survival compared with wild-type mice after MI.^64^

In summary, circadian rhythms exhibit a clear influence on infarction damage; yet, the interplay and relevance of oscillations in immune cells and the vasculature in this setting remain to be further determined.

## CONCLUSIONS AND PERSPECTIVES

Circadian rhythms regulate the function of both the vasculature and the immune system. In both steady state and disease, adhesion molecules on leukocytes and blood and lymph vessels show a time-of-day–dependent expression pattern, which contributes to a time-of-day dependency in immune cell trafficking (Figure 1; Table 1). Immune cells play important roles in the cause and damage of vascular diseases, within and outside the vasculature.^157,158^ Oscillations in monocyte and neutrophil adhesion to vascular beds are highly associated with the onset and severity of atherosclerosis^111^ and MI.^35,150^ However, the interplay between the vasculature and leukocytes is often overlooked, with little causal relationship in studies of atherosclerosis and infarction. Furthermore, single-cell RNA sequencing has revealed that ECs are highly heterogeneous with respect to caliber and vascular beds.^159,160^ The heart (among many other organs and tissues) is populated by tissue-resident macrophages at a steady state,^161^ which can instruct the infiltration and differentiation of blood monocytes after MI.^161–164^ The distinction of how tissue-resident macrophages respond to time-of-day MI in comparison to monocytes recruited from the blood remains to be investigated. Therefore, more focus should be placed on the mutual interactions between ECs and immune cells in a subset- and tissue-specific manner, including, but not limited to, adhesion and migration, in circadian cardiovascular biology.

In general, disturbances in circadian rhythms worsen the conditions of atherosclerosis and heart attacks, with various circadian clock genes participating in their regulation. However, different manipulation methods, target cells, and models may generate conflicting results (Figure 2; Table 2), which can be due to the specific disease model used and whether constitutive or induced circadian clock gene knockouts were assessed. Inducible circadian clock gene manipulation models should be preferred to avoid any interferences of blood vessels and immune systems during the development and maturation of organisms.

Many findings generated in preclinical mouse models do not translate into humans. For example, the association between the time-of-day at symptom onset and the severity of MI has been unequivocal in mice^35,132,148–153^ but controversial in patients.^140–144^ Given that mice are nocturnal and humans are diurnal, using other—diurnal—animal models to study the circadian rhythms in cardiovascular diseases might be beneficial in aiding the translational potential of studies. Although several approaches have been proposed to target circadian rhythms in cardiovascular diseases^30^ and because retrospective studies found a time-of-day difference in the efficacy of immune checkpoint inhibitors,^165–167^ prospective clinical studies are required to validate the efficacy of chronotherapies for vascular diseases.

One can envision that circadian rhythms could be harnessed to optimally treat cardiovascular diseases. A simple biomarker in blood, namely, the number of leukocytes, exhibits a 2- to 3-fold difference in their abundance. Furthermore, platelet function and interactions with the blood vasculature exhibit time-of-day differences,^81,99^ pointing toward different levels of effectiveness of thrombolytic reagents across the day. While some preclinical studies show time-of-day effectiveness in the administration of antiatherosclerotic drugs,^168^ clinical trials (NCT00725127 and NCT05932472) testing the protective effect of morning versus bedtime aspirin (an anticoagulant drug) intake from major cardiovascular events are ongoing.

Chronotherapies targeting vascular-immune interactions could be a promising tool to treat cardiovascular diseases. Of note, in a preclinical mouse atherosclerosis model, daily intraperitoneal administration of a CCR2 antagonist, RS102895, 8 hours before the peak (ZT1) of monocyte adhesion to the carotid artery, was shown to decrease the atherosclerotic plaque size by 40%.^111^ Furthermore, 2 preclinical studies proposed chemokine receptor antagonists (the CCR2 inhibitor RS504393^150^ and the CXCR2 inhibitor SB225002^35^) to be protective against severe MI damage at ZT13, leading to reduced infiltration of Ly6C^+^ monocytes and CXCR2^+^ neutrophils, respectively, to the heart on MI. REV-ERBα could be another promising target for chronotherapy. Activation by daily intraperitoneal injection of the agonist SR9009 was shown to lower atherosclerotic plaque size by 23% in mice.^121^ Moreover, in another elegantly designed study, SR9009 was shown to alleviate MI damage by ≈30% in both mice and sheep, via a reduction in both neutrophil infiltration and inflammatory cytokine expression.^134^ To date, no clinical chronotherapeutic studies have been approved for the treatment of vascular diseases, but these molecules might be a promising way to follow for future clinical trials in the context of atherosclerosis and MI. Furthermore, clinical and epidemiological studies have suggested that taking antihypertensive medications at bedtime could enhance the nighttime blood pressure profile. In different placebo-controlled clinical trials across different countries, evening dosing of antihypertensive medication was associated with a reduced risk of cardiovascular outcomes.^169^ These findings need to be replicated in prospective, controlled, multicenter trials.

Lately, increasing attention is also focusing on how the timing of food intake affects blood pressure, particularly with respect to nocturnal hypertension. Current evidence suggests a connection between eating at night and a higher risk of elevated blood pressure and disrupted circadian rhythms.^170^ Furthermore, numerous studies have highlighted the cardiometabolic advantages of time-restricted feeding in humans.^171^ In animal studies, mice with food access limited to their light (ie, rest) phase showed metabolic impairments, inverted blood pressure rhythms,^172^ and disrupted microbiome that impairs cardiac repair.^126^ Restricting food intake times to a specific daily interval has become a popular way to improve metabolic health,^173^ and it might, therefore, be exploited in the future as a noninvasive method to prevent the onset of cardiovascular diseases.

In conclusion, time-of-day plays a critical role in regulating the vascular-immune interaction under both healthy conditions and vascular diseases. Disturbing circadian rhythms using various techniques or in specific cell types may yield distinct outcomes in vascular diseases. Further investigation is warranted to unravel the underlying mechanisms, with a particular emphasis on understanding the intricate mutual interactions between the vascular and immune systems.

## ARTICLE INFORMATION

### Acknowledgments

Figures were created with BioRender.com.

### Sources of Funding

The work in the Scheiermann Laboratory was supported by grants from the European Research Council (CoG 101001233 and CIRCADYN to C. Scheiermann), the Swiss National Science Foundation (310030_219256/1 to C. Scheiermann), the Swiss Cancer League (KLS-4836-08-2019 to C. Scheiermann), and the Geneva Cancer League (2106 to C. Scheiermann).

### Disclosures

None.
